# Supplementary material for: Molecular profiling and phenotypic evaluation of thermo-sensitive genic male sterility genes for high-yielding rice hybrids (Oryza sativa L.)
Source: PeerJ. 2025 Mar 26;13:e18803. doi: 10.7717/peerj.18803 (PMC11954467; doi:10.7717/peerj.18803)
Supplement: Supplemental Information 1 — ** statistically significant at p = 0.05, implying that the observed differences in the traits between genotypes are statistically significant and unlikely to be due to random variation. [file peerj-13-18803-s001.docx]

**Table 3.** Analysis of variance for yield and floral traits of TGMS lines

| **S. No.** | **Source of variation** | **Genotype** | **Replication** | **Error** |
| --- | --- | --- | --- | --- |
| 1 | Degrees of freedom | 56 | 2 | 112 |
| 2 | Days to 50% flowering | 650.90** | 15.88 | 33.42 |
| 3 | Plant height (cm) | 163.58** | 17.65 | 18.96 |
| 4 | Number of productive tillers per plant | 35.37** | 0.22 | 0.91 |
| 5 | Panicle exertion (%) | 77.80** | 23.16 | 11.57 |
| 6 | Panicle length (cm) | 17.88** | 2.12 | 1.51 |
| 7 | Number of grains per panicle (g) | 8350.28** | 89.15 | 52.53 |
| 8 | Single plant yield (g) | 229.31** | 4.94 | 1.97 |
| 9 | Pollen sterility (%) | 1482.21** | 4.56 | 17.76 |
| 10 | Pollen fertility (%) | 888.83** | 3.59 | 10.28 |
| 11 | Spikelet ferility | 196.63** | 14.84 | 7.64 |
| 12 | Stigma length (mm) | 0.20** | 0.01 | 0.01 |
| 13 | Stigma exertion (%) | 368.57** | 27.10 | 3.84 |
| 14 | Glume angle (^0^) | 117.44** | 0.42 | 1.60 |
|  | | | | |

** statistically significant at **p = 0.05**, implying that the observed differences in the traits between genotypes are statistically significant and unlikely to be due to random variation.
