# Supplementary material for: Molecular profiling and phenotypic evaluation of thermo-sensitive genic male sterility genes for high-yielding rice hybrids (Oryza sativa L.)
Source: PeerJ. 2025 Mar 26;13:e18803. doi: 10.7717/peerj.18803 (PMC11954467; doi:10.7717/peerj.18803)
Supplement: Supplemental Information 2 [file peerj-13-18803-s002.docx]

**Supplementary table 2.** Mean biometrical data of TGMS lines at HREC, Gudalur

| **S. No** | **Entry** | **Days to 50% flowering** | **Plant height(cm)** | **Productive tillers per plant** | **Panicle exertion (%)** | | **Panicle length (cm)** | **Number of grains per panicle** | **Pollen fertility** | | **Spikelet fertility percentage (%)** | | **Single plant yield (g)** |
| --- | --- | --- | --- | --- | --- | --- | --- | --- | --- | --- | --- | --- | --- |
|  |  |  |  |  | **Panicle exertion** | **Transformed values** |  |  | **Pollen fertility (%)** | **Transformed value** | **Spikelet ferilty** | **Transformed values** |  |
| 1 | TNAU 1S | 115 | 94.67 | 17.33 | 79.2 | 63 | 25.33 | 216 | 56 | 48.5 | 60.3 | 50.9 | 16.5 |
| 2 | TNAU 2S | 97 | 93 | 17 | 86.3 | 68.7 | 25 | 106 | 93.6 | 76 | 63.1 | 52.6 | 11 |
| 3 | TNAU 4S | 94 | 84.33 | 18.33 | 80.2 | 64.1 | 23 | 237 | 88.4 | 70.1 | 73.2 | 58.9 | 32.5 |
| 4 | TNAU 4S-1 | 99 | 72.33 | 15.67 | 79.1 | 62.9 | 21 | 129 | 66.8 | 54.8 | 67.7 | 55.4 | 26.8 |
| 5 | TNAU 15S | 72.3 | 83.9 | 14.2 | 82.9 | 66.1 | 18.3 | 260 | 94.7 | 76.8 | 86.1 | 68.2 | 11.5 |
| 6 | TNAU 16S | 91 | 66.67 | 27.67 | 100 | 89.7 | 21.67 | 131 | 97.1 | 80.3 | 82.9 | 65.6 | 33.8 |
| 7 | TNAU 18S | 94 | 68 | 20.67 | 77.8 | 61.9 | 19 | 218 | 64.4 | 53.4 | 44.4 | 41.8 | 28.7 |
| 8 | TNAU 19S | 108 | 80 | 22.33 | 81.3 | 64.8 | 22.67 | 173 | 97 | 80.1 | 75.1 | 60.1 | 37.5 |
| 9 | TNAU 23S | 114 | 76.33 | 17.33 | 85.1 | 67.5 | 20.33 | 128 | 93.9 | 75.8 | 71.3 | 57.6 | 18.7 |
| 10 | TNAU 30S | 117 | 85.67 | 13.33 | 74.9 | 60.5 | 23 | 216 | 92.2 | 73.8 | 56.1 | 48.5 | 26.2 |
| 11 | TNAU 31S | 101 | 78 | 21.33 | 78 | 63.2 | 21.67 | 130 | 55.8 | 48.3 | 24.8 | 57.8 | 24.2 |
| 12 | TNAU 34S | 101 | 79 | 22.33 | 76.8 | 61.3 | 24.67 | 129 | 75.7 | 60.4 | 43.9 | 72.2 | 41 |
| 13 | TNAU 37S | 97 | 70.67 | 21.33 | 73.5 | 59.6 | 19.33 | 121 | 92.1 | 74.5 | 67.6 | 55.3 | 40.2 |
| 14 | TNAU 38S | 114 | 93.33 | 22 | 79.4 | 63.1 | 24.67 | 96 | 62.7 | 52.4 | 26.2 | 79.2 | 21.2 |
| 15 | TNAU 39S | 86 | 85.33 | 17 | 76.2 | 61 | 23 | 183 | 83.3 | 65.9 | 71.9 | 58 | 16.5 |
| 16 | TNAU 45S | 114 | 77.33 | 11.33 | 72.9 | 58.9 | 21 | 224 | 93.1 | 74.8 | 69.8 | 56.7 | 23.2 |
| 17 | TNAU 50S | 108 | 78.33 | 14.67 | 80.9 | 64.7 | 22.67 | 127 | 94.6 | 76.5 | 64.1 | 53.2 | 19.2 |
| 18 | TNAU 51S | 108 | 82 | 19.67 | 72 | 62.1 | 22.33 | 132 | 95.6 | 77.9 | 76.8 | 61.2 | 39.7 |
| 19 | TNAU 53S | 112 | 70.67 | 20.33 | 81 | 64.3 | 24.67 | 188 | 95.2 | 77.5 | 69 | 56.2 | 19 |
| 20 | TNAU 59S-1 | 109 | 77.67 | 17.33 | 78.6 | 63.7 | 22.67 | 104 | 95 | 77.1 | 78.5 | 62.4 | 32 |
| 21 | TNAU 59S-2 | 107 | 71.33 | 16 | 81.8 | 65.7 | 23 | 128 | 93.1 | 74.8 | 68.3 | 55.7 | 25.5 |
| 22 | TNAU 60S | 138 | 81.33 | 14 | 77.3 | 61.7 | 23.33 | 135 | 89.1 | 70.7 | 59.1 | 50.3 | 31.3 |
| 23 | TNAU 71S | 116 | 89 | 15.67 | 75.1 | 60.2 | 22.67 | 181 | 94.2 | 76 | 40 | 39.2 | 30 |
| 24 | TNAU 82S | 111 | 76 | 14.67 | 78.3 | 63.1 | 20.33 | 106 | 96.8 | 79.8 | 82.3 | 65.1 | 35.7 |
| 25 | TNAU 83S | 108 | 80.67 | 18.33 | 81.4 | 64.5 | 19.33 | 170 | 95.2 | 77.4 | 45.9 | 42.6 | 22.7 |
| 26 | TNAU 85S | 101 | 81.33 | 18.33 | 75.1 | 60.2 | 21 | 135 | 95.5 | 77.7 | 60.5 | 51.1 | 19 |
| 27 | TNAU 86S | 108 | 80.67 | 15.33 | 80.7 | 64.3 | 22 | 229 | 88.6 | 70.2 | 80.3 | 63.7 | 19.5 |
| 28 | TNAU 92S | 96 | 83.33 | 19 | 71.6 | 58 | 19.67 | 202 | 94.7 | 76.8 | 33.3 | 35.3 | 10 |
| 29 | TNAU 93S | 91 | 61.67 | 21.33 | 83.4 | 67 | 15 | 96 | 31.9 | 7.9 | 16.6 | 43.1 | 30.7 |
| 30 | TNAU 95S | 112 | 80.33 | 25 | 73.8 | 59.2 | 20.33 | 186 | 98.6 | 83.1 | 73.9 | 59.3 | 24 |
| 31 | TNAU 98S | 124 | 91.67 | 20.33 | 83.2 | 65.9 | 24.67 | 176 | 95.7 | 78 | 69.8 | 56.7 | 28.5 |
| 32 | TNAU 100S | 126 | 86.33 | 15.33 | 83.6 | 67.5 | 23.67 | 147 | 85.5 | 67.6 | 59.8 | 50.7 | 19.3 |
| 33 | TNAU 101S | 102 | 71.67 | 14.33 | 69.6 | 56.6 | 22.33 | 154 | 95.5 | 77.7 | 60.8 | 51.2 | 7.7 |
| 34 | TNAU 102S | 97 | 83.67 | 15.67 | 77.3 | 62.3 | 24.67 | 108 | 95.5 | 77.7 | 60.5 | 51.1 | 21.2 |
| 35 | TNAU 103S | 97 | 84.67 | 13 | 78.4 | 63.1 | 26.33 | 148 | 92.7 | 74.7 | 72.8 | 58.6 | 23.7 |
| 36 | TNAU 106S | 112 | 84 | 18.33 | 80.1 | 64 | 15.67 | 200 | 94 | 75.9 | 79.8 | 63.3 | 26.2 |
| 37 | TNAU 107S | 152 | 76.67 | 22.33 | 80 | 64.8 | 20.67 | 198 | 93.9 | 75.8 | 73.5 | 59 | 14.2 |
| 38 | TNAU 111S | 152 | 82 | 22 | 81.4 | 64.5 | 23 | 161 | 50.8 | 45.5 | 34.4 | 53.4 | 30.5 |
| 39 | TNAU 112S | 62.3 | 92.5 | 15.9 | 86.6 | 68.5 | 19.8 | 34 | 50.6 | 45.4 | 27.5 | 69.3 | 21.8 |
| 40 | TNAU 113S | 108 | 80 | 22.33 | 73.2 | 59 | 17.67 | 321 | 51.5 | 7 | 14.7 | 53.6 | 36.3 |
| 41 | TNAU 114S | 112 | 78.33 | 16 | 73.3 | 60.4 | 18.67 | 326 | 93.9 | 75.7 | 62.6 | 52.3 | 43 |
| 42 | TNAU 115S | 102 | 90.33 | 19.33 | 65.2 | 54 | 25 | 185 | 84.6 | 67 | 78.9 | 62.7 | 16 |
| 43 | TNAU 115S-1 | 119 | 79.33 | 21.33 | 65.2 | 54 | 23.33 | 185 | 84.6 | 67 | 78.9 | 62.7 | 16 |
| 44 | TNAU 116S | 111 | 85 | 18.67 | 77.4 | 61.9 | 20 | 143 | 64.9 | 44 | 45 | 55.8 | 14.8 |
| 45 | TNAU 120S | 102 | 84 | 17 | 87.6 | 69.4 | 20 | 124 | 76.6 | 61.1 | 62.8 | 52.8 | 18.5 |
| 46 | TNAU 126S-1 | 101 | 89.33 | 21.33 | 88.4 | 70.3 | 22.67 | 135 | 95.9 | 78.4 | 84.4 | 66.8 | 25 |
| 47 | TNAU 126S-2 | 108 | 90 | 14.33 | 88.4 | 70.3 | 22.33 | 135 | 95.9 | 78.4 | 84.4 | 66.8 | 25 |
| 48 | TNAU 127S | 112 | 86.33 | 20.67 | 76.1 | 61.2 | 24.33 | 140.00 | 94.4 | 76.3 | 52.6 | 45.6 | 16.00 |
| 49 | TNAU 129S | 99 | 87.67 | 17.67 | 78.4 | 62.4 | 20 | 209 | 95.7 | 78.1 | 64.1 | 53.2 | 14.3 |
| 50 | TNAU 131S | 98 | 82.33 | 15.33 | 75.7 | 60.5 | 18.67 | 138 | 92.2 | 73.8 | 69.1 | 56.3 | 42.3 |
| 51 | TNAU 132S | 108 | 87.33 | 10 | 72.3 | 58.5 | 22.67 | 140 | 94.4 | 76.3 | 52.6 | 45.6 | 16 |
| 52 | TNAU 135S | 118 | 84.33 | 14.67 | 77.1 | 61.9 | 21.33 | 191 | 96.6 | 79.5 | 71.1 | 57.5 | 28.3 |
| 53 | TNAU 136S | 94 | 73.67 | 19.33 | 86.6 | 69 | 18.33 | 160 | 63.3 | 14.8 | 62.3 | 52.1 | 26.8 |
| 54 | TNAU 137S-1 | 110 | 90.67 | 18.67 | 74.7 | 60.2 | 22 | 184 | 66.7 | 54.8 | 74.1 | 59.4 | 28.3 |
| 55 | TNAU 137S-2 | 108 | 89 | 15.67 | 74.7 | 60.2 | 22.67 | 184 | 66.7 | 54.8 | 74.1 | 59.4 | 28.3 |
| 56 | TNAU 142S | 102 | 96.67 | 14 | 73 | 58.7 | 26 | 232 | 94.9 | 77 | 63.7 | 53 | 36.3 |
| 57 | TNAU 143S | 102 | 80.33 | 18.33 | 81.5 | 64.9 | 21 | 144 | 94.7 | 76.8 | 67 | 54.9 | 31.5 |
| MIN |  | 62.3 | 61.67 | 10 |  | 54 | 15 | 34 |  | 7 |  | 35.3 | 7.7 |
| MAX |  | 152 | 96.67 | 27.67 |  | 89.7 | 26.33 | 326 |  | 83.1 |  | 79.2 | 43 |
| MEAN |  | 106.62 | 81.94 | 17.91 |  | 63.32 | 21.76 | 165.68 |  | 67.16 |  | 56.16 | 25.14 |
| SEM |  | 4.72 | 3.56 | 0.78 |  | 2.78 | 1 | 5.91 |  | 2.62 |  | 2.26 | 1.14 |
| MEAN+SEM |  | 111.34 | 85.50 | 18.69 |  | 66.10 | 22.76 | 171.59 |  | 69.78 |  | 58.42 | 26.28 |
| MEAN-SEM |  | 101.90 | 78.38 | 17.13 |  | 60.54 | 20.76 | 159.77 |  | 64.54 |  | 53.90 | 24.00 |
