# Supplementary material for: Molecular profiling and phenotypic evaluation of thermo-sensitive genic male sterility genes for high-yielding rice hybrids (Oryza sativa L.)
Source: PeerJ. 2025 Mar 26;13:e18803. doi: 10.7717/peerj.18803 (PMC11954467; doi:10.7717/peerj.18803)
Supplement: Supplemental Information 3 [file peerj-13-18803-s003.docx]

**Supplementary table 3.** Mean biometrical data of TGMS lines at Coimbatore.

| **S. No.** | **Entry** | **Days to 50% flowering** | **Plant height(cm)** | **Pollen sterility (%)** | | **Stigma length (mm)** | **Stigma exertion (%)** | | **Glume angle(°)** |
| --- | --- | --- | --- | --- | --- | --- | --- | --- | --- |
|  |  |  |  | **Pollen sterility** | **Transformed values** |  | **Stigma exertion** | **Transformed values** |  |
| 1 | TNAU 1S | 100 | 85.2 | 100 | 89.7 | 1.51 | 53.1 | 46.8 | 23.3 |
| 2 | TNAU 2S | 107 | 74.67 | 100 | 89.7 | 1.47 | 48.3 | 44.1 | 30 |
| 3 | TNAU 4S | 89 | 79.17 | 100 | 89.7 | 2.22 | 53.9 | 47.2 | 11.7 |
| 4 | TNAU 4S-1 | 99 | 84.33 | 100 | 89.7 | 1.75 | 32.3 | 56.6 | 13.3 |
| 5 | TNAU 15S | 126 | 73.67 | 100 | 89.7 | 1.54 | 21.8 | 27.8 | 28.3 |
| 6 | TNAU 16S | 113 | 84.3 | 100 | 89.7 | 1.65 | 32.8 | 35 | 28.3 |
| 7 | TNAU 18S | 111 | 77 | 100 | 89.7 | 1.56 | 70.2 | 50.2 | 11.7 |
| 8 | TNAU 19S | 105 | 82 | 100 | 89.7 | 1.83 | 70.2 | 56.9 | 25 |
| 9 | TNAU 23S | 113 | 86 | 100 | 89.7 | 2.1 | 20 | 26.6 | 16.7 |
| 10 | TNAU 30S | 105 | 81 | 8.2 | 15.6 | 2.07 | 19.3 | 26 | 23.3 |
| 11 | TNAU 31S | 103 | 64.67 | 100 | 89.7 | 2.02 | 39 | 38.7 | 13.3 |
| 12 | TNAU 34S | 102 | 89 | 100 | 89.7 | 1.51 | 35.7 | 36.7 | 21.7 |
| 13 | TNAU 37S | 94 | 75.67 | 100 | 89.7 | 1.7 | 53.1 | 46.8 | 28.3 |
| 14 | TNAU 38S | 115 | 94.5 | 100 | 89.7 | 1.93 | 43.6 | 41.3 | 11.7 |
| 15 | TNAU 39S | 96 | 86 | 100 | 89.7 | 1.92 | 70.8 | 57.3 | 25 |
| 16 | TNAU 45S | 115 | 88 | 100 | 89.7 | 1.96 | 14.8 | 22.4 | 13.3 |
| 17 | TNAU 50S | 126 | 104.5 | 100 | 89.7 | 1.9 | 32.3 | 34.6 | 23.3 |
| 18 | TNAU 51S | 113 | 81.17 | 100 | 89.7 | 1.92 | 33.7 | 35.5 | 23.3 |
| 19 | TNAU 53S | 111 | 85.33 | 93.4 | 17.2 | 1.48 | 57.1 | 49.1 | 16.7 |
| 20 | TNAU 59S-1 | 107 | 75.67 | 100 | 89.7 | 1.49 | 49.7 | 44.9 | 11.7 |
| 21 | TNAU 59S-2 | 109 | 75.86 | 100 | 89.7 | 1.75 | 40 | 39.2 | 18.3 |
| 22 | TNAU 60S | 105 | 70.33 | 100 | 89.7 | 1.78 | 41.1 | 39.9 | 20 |
| 23 | TNAU 71S | 112 | 90.2 | 100 | 89.7 | 1.2 | 23.9 | 29.3 | 16.7 |
| 24 | TNAU 82S | 107 | 75.83 | 24.3 | 29.4 | 1.73 | 31.9 | 46 | 15 |
| 25 | TNAU 83S | 107 | 78.17 | 100 | 89.7 | 2.05 | 51.7 | 43.4 | 18.3 |
| 26 | TNAU 85S | 102 | 74.67 | 100 | 89.7 | 2.19 | 75 | 34.4 | 28.01 |
| 27 | TNAU 86S | 111 | 77 | 23.4 | 28.7 | 2.18 | 27.1 | 31.4 | 11.7 |
| 28 | TNAU 92S | 113 | 74.33 | 100 | 89.7 | 2.09 | 84.6 | 66.9 | 28.3 |
| 29 | TNAU 93S | 113 | 76.67 | 34.8 | 36.1 | 1.7 | 41.7 | 40.2 | 18.3 |
| 30 | TNAU 95S | 103 | 68.67 | 100 | 89.7 | 1.83 | 26.7 | 31.1 | 20 |
| 31 | TNAU 98S | 111 | 77.67 | 100 | 89.7 | 1.93 | 10.9 | 19.3 | 28.3 |
| 32 | TNAU 100S | 102 | 78 | 100 | 89.7 | 2.32 | 28.4 | 32.2 | 25 |
| 33 | TNAU 101S | 100 | 80 | 100 | 89.7 | 1.9 | 21.8 | 34.2 | 20 |
| 34 | TNAU 102S | 103 | 82 | 100 | 89.7 | 1.7 | 38.2 | 38.2 | 11.7 |
| 35 | TNAU 103S | 105 | 75 | 100 | 89.7 | 1.91 | 30 | 33.2 | 21.7 |
| 36 | TNAU 106S | 115 | 79.67 | 100 | 89.7 | 1.83 | 35.3 | 36.5 | 21.7 |
| 37 | TNAU 107S | 116 | 80.23 | 100 | 89.7 | 1.88 | 37.3 | 37.6 | 16.7 |
| 38 | TNAU 111S | 97 | 109 | 100 | 89.7 | 1.78 | 26.8 | 31.2 | 11.7 |
| 39 | TNAU 112S | 99 | 110.33 | 100 | 89.7 | 2.44 | 85.9 | 68 | 21.7 |
| 40 | TNAU 113S | 99 | 111 | 11.7 | 89.7 | 2.48 | 23.8 | 29.2 | 20 |
| 41 | TNAU 114S | 113 | 77.6 | 92.8 | 19.3 | 2.08 | 49.1 | 44.5 | 25 |
| 42 | TNAU 115S | 102 | 92 | 92.8 | 74.8 | 1.93 | 31.5 | 34.1 | 28.3 |
| 43 | TNAU 115S-1 | 120 | 80 | 100 | 89.7 | 1.93 | 31.5 | 34.1 | 28.3 |
| 44 | TNAU 116S | 109 | 87 | 100 | 89.7 | 1.89 | 54 | 47.3 | 13.3 |
| 45 | TNAU 120S | 104 | 87 | 100 | 89.7 | 1.6 | 29.5 | 23.9 | 21.7 |
| 46 | TNAU 126S-1 | 141 | 68.33 | 100 | 89.7 | 2.18 | 24.1 | 27.6 | 31.7 |
| 47 | TNAU 126S-2 | 110 | 92 | 100 | 89.7 | 2.18 | 24.1 | 27.6 | 31.7 |
| 48 | TNAU 127S | 110 | 88 | 100 | 89.7 | 1.49 | 41.66 | 40.1 | 14.7 |
| 49 | TNAU 129S | 127 | 60 | 100 | 13.8 | 1.89 | 53.6 | 47.1 | 11.7 |
| 50 | TNAU 131S | 104 | 65 | 100 | 89.7 | 1.86 | 24.7 | 29.8 | 21.7 |
| 51 | TNAU 132S | 107 | 86 | 100 | 89.7 | 2.18 | 70.5 | 57.1 | 16.7 |
| 52 | TNAU 135S | 118 | 71.67 | 100 | 89.7 | 1.75 | 33.7 | 35.5 | 10 |
| 53 | TNAU 136S | 105 | 86.83 | 95.5 | 89.7 | 2.01 | 60 | 50.8 | 21.7 |
| 54 | TNAU 137S-1 | 108 | 92 | 95.5 | 78.3 | 2.1 | 63.5 | 52.9 | 13.3 |
| 55 | TNAU 137S-2 | 106 | 90 | 91.4 | 78.3 | 2.1 | 63.5 | 52.9 | 13.3 |
| 56 | TNAU 142S | 102 | 98 | 100 | 73.2 | 1.96 | 76.6 | 61.1 | 18.3 |
| 57 | TNAU 143S | 104 | 82 | 100 | 89.7 | 1.88 | 48.7 | 44.3 | 28.3 |
| **MIN** |  | 89 | 60 |  | 13.8 | 1.2 |  | 19.3 | 10 |
| **MAX** |  | 141 | 111 |  | 89.7 | 2.48 |  | 68 | 31.7 |
| **MEAN** |  | 109.84 | 82.45 |  | 80.54 | 1.88 |  | 40.29 | 20.05 |
| **SEM** |  | 1.17 | 1.4 |  | 3.44 | 0.08 |  | 1.6 | 1.03 |
| **MEAN+SE** |  | 111.01 | 83.85 |  | 83.98 | 1.96 |  | 41.89 | 21.08 |
| **MEAN-SE** |  | 108.67 | 81.05 |  | 77.10 | 1.80 |  | 38.69 | 19.02 |
