# Supplementary material for: Molecular profiling and phenotypic evaluation of thermo-sensitive genic male sterility genes for high-yielding rice hybrids (Oryza sativa L.)
Source: PeerJ. 2025 Mar 26;13:e18803. doi: 10.7717/peerj.18803 (PMC11954467; doi:10.7717/peerj.18803)
Supplement: Supplemental Information 4 [file peerj-13-18803-s004.docx]

Supplementary Table 4. Molecular profiling of TGMS lines

| **S. No.** | **Entry** | **tms 4** | **tms 5** | | | | **tms 8** | | **tms 10** | **Pollen sterility (%)** |
| --- | --- | --- | --- | --- | --- | --- | --- | --- | --- | --- |
|  |  | **RM27** | **RM174** | **RM5897** | **RM6247** | **RM7575** | **RM21** | **RM224** | **RM300** |  |
| 1 | TNAU 1S | 0 | 1 | 1 | 1 | 0 | 1 | 1 | 1 | 100 |
| 2 | TNAU 2S | 0 | 1 | 1 | 1 | 0 | 1 | 1 | 1 | 100 |
| 3 | TNAU 4S | 0 | 1 | 1 | 1 | 0 | 1 | 1 | 0 | 100 |
| 4 | TNAU 4S-1 | 0 | 0 | 1 | 1 | 0 | 1 | 1 | 0 | 100 |
| 5 | TNAU 15S | 0 | 0 | 1 | 1 | 0 | 1 | 1 | 1 | 100 |
| 6 | TNAU 16S | 0 | 1 | 1 | 1 | 0 | 0 | 1 | 0 | 100 |
| 7 | TNAU 18S | 0 | 1 | 1 | 0 | 1 | 1 | 1 | 1 | 100 |
| 8 | TNAU 19S | 0 | 1 | 1 | 0 | 1 | 1 | 1 | 1 | 100 |
| 9 | TNAU 23S | 1 | 0 | 1 | 0 | 1 | 1 | 1 | 1 | 100 |
| 10 | TNAU 30S | 0 | 1 | 1 | 0 | 1 | 1 | 1 | 1 | 8.2 |
| 11 | TNAU 31S | 0 | 0 | 1 | 0 | 1 | 1 | 1 | 1 | 100 |
| 12 | TNAU 34S | 0 | 0 | 1 | 0 | 1 | 1 | 1 | 1 | 100 |
| 13 | TNAU 37S | 0 | 0 | 1 | 0 | 1 | 1 | 1 | 1 | 100 |
| 14 | TNAU 38S | 1 | 1 | 1 | 1 | 1 | 1 | 1 | 1 | 100 |
| 15 | TNAU 39S | 1 | 1 | 0 | 1 | 1 | 1 | 1 | 1 | 100 |
| 16 | TNAU 45S | 1 | 0 | 1 | 1 | 1 | 1 | 1 | 0 | 100 |
| 17 | TNAU 50S | 0 | 0 | 1 | 1 | 1 | 0 | 1 | 0 | 100 |
| 18 | TNAU 51S | 0 | 0 | 1 | 1 | 1 | 1 | 1 | 1 | 100 |
| 19 | TNAU 53S | 0 | 0 | 1 | 1 | 1 | 0 | 1 | 0 | 93.4 |
| 20 | TNAU 59S-1 | 0 | 1 | 1 | 1 | 1 | 1 | 1 | 1 | 100 |
| 21 | TNAU 59S-2 | 0 | 1 | 0 | 1 | 1 | 0 | 1 | 1 | 100 |
| 22 | TNAU 60S | 1 | 1 | 1 | 1 | 1 | 1 | 1 | 0 | 100 |
| 23 | TNAU 71S | 0 | 0 | 1 | 1 | 1 | 0 | 1 | 1 | 100 |
| 24 | TNAU 82S | 0 | 1 | 1 | 1 | 1 | 1 | 1 | 1 | 24.3 |
| 25 | TNAU 83S | 0 | 1 | 1 | 1 | 1 | 1 | 1 | 1 | 100 |
| 26 | TNAU 85S | 0 | 1 | 1 | 1 | 1 | 1 | 1 | 1 | 100 |
| 27 | TNAU 86S | 0 | 1 | 1 | 1 | 1 | 1 | 1 | 0 | 23.4 |
| 28 | TNAU 92S | 0 | 1 | 1 | 1 | 1 | 1 | 1 | 0 | 100 |
| 29 | TNAU 93S | 0 | 1 | 1 | 1 | 1 | 1 | 1 | 0 | 34.8 |
| 30 | TNAU 95S | 0 | 1 | 1 | 0 | 0 | 1 | 1 | 0 | 100 |
| 31 | TNAU 98S | 0 | 1 | 0 | 1 | 0 | 1 | 1 | 1 | 100 |
| 32 | TNAU 100S | 0 | 1 | 1 | 0 | 0 | 1 | 1 | 0 | 100 |
| 33 | TNAU 101S | 0 | 0 | 1 | 0 | 0 | 0 | 1 | 0 | 100 |
| 34 | TNAU 102S | 0 | 0 | 0 | 0 | 0 | 1 | 1 | 1 | 100 |
| 35 | TNAU 103S | 0 | 1 | 1 | 0 | 0 | 0 | 1 | 0 | 100 |
| 36 | TNAU 106S | 0 | 1 | 1 | 0 | 0 | 1 | 1 | 1 | 100 |
| 37 | TNAU 107S | 0 | 1 | 1 | 0 | 0 | 1 | 1 | 1 | 100 |
| 38 | TNAU 111S | 0 | 1 | 1 | 0 | 0 | 1 | 1 | 1 | 100 |
| 39 | TNAU 112S | 0 | 1 | 1 | 0 | 0 | 1 | 1 | 0 | 100 |
| 40 | TNAU 113S | 0 | 1 | 1 | 0 | 0 | 1 | 1 | 0 | 11.7 |
| 41 | TNAU 114S | 0 | 0 | 1 | 0 | 0 | 1 | 1 | 0 | 92.8 |
| 42 | TNAU 115S | 0 | 1 | 1 | 0 | 0 | 0 | 1 | 0 | 92.8 |
| 43 | TNAU 115S-1 | 0 | 1 | 1 | 0 | 0 | 0 | 1 | 0 | 100 |
| 44 | TNAU 116S | 0 | 1 | 1 | 0 | 0 | 0 | 1 | 0 | 100 |
| 45 | TNAU 120S | 0 | 0 | 1 | 0 | 0 | 0 | 1 | 1 | 100 |
| 46 | TNAU 126S-1 | 0 | 0 | 1 | 0 | 0 | 0 | 1 | 1 | 100 |
| 47 | TNAU 126S-2 | 0 | 0 | 1 | 0 | 0 | 0 | 1 | 1 | 100 |
| 48 | TNAU 127S | 0 | 1 | 1 | 0 | 0 | 0 | 1 | 1 | 100 |
| 49 | TNAU 129S | 0 | 0 | 1 | 0 | 0 | 0 | 1 | 0 | 100 |
| 50 | TNAU 131S | 0 | 1 | 1 | 0 | 0 | 1 | 1 | 1 | 100 |
| 51 | TNAU 132S | 0 | 1 | 1 | 0 | 0 | 1 | 1 | 0 | 100 |
| 52 | TNAU 135S | 0 | 1 | 1 | 0 | 0 | 0 | 1 | 0 | 100 |
| 53 | TNAU 136S | 0 | 1 | 1 | 0 | 0 | 1 | 1 | 0 | 95.5 |
| 54 | TNAU 137S-1 | 0 | 1 | 1 | 0 | 0 | 0 | 1 | 0 | 95.5 |
| 55 | TNAU 137S-2 | 0 | 0 | 1 | 0 | 0 | 1 | 1 | 1 | 91.4 |
| 56 | TNAU 142S | 0 | 1 | 1 | 0 | 0 | 1 | 1 | 1 | 100 |
| 57 | TNAU 143S | 0 | 0 | 0 | 0 | 0 | 1 | 1 | 1 | 100 |
