# Supplementary material for: Molecular profiling and phenotypic evaluation of thermo-sensitive genic male sterility genes for high-yielding rice hybrids (Oryza sativa L.)
Source: PeerJ. 2025 Mar 26;13:e18803. doi: 10.7717/peerj.18803 (PMC11954467; doi:10.7717/peerj.18803)
Supplement: Supplemental Information 5 — PS, Pollen Sterility (%); PE, Panicle Exertion (%); SL, Stigma Length (mm); SE, Stigma Exertion (%); GA, Glume Angle (°); SPY, Single Plant Yield (g); N, Normalised values. [file peerj-13-18803-s005.docx]

Supplementary table 5. Ranking of TGMS Genotypes Based on the Composite Scores for Traits Influencing Outcrossing Efficiency.

| **Ranking** | **Line** | **Gene Combination** | **PE** | | **SL** | | **SE** | | **GA** | | **SPY** | | **Composite Score** |
| --- | --- | --- | --- | --- | --- | --- | --- | --- | --- | --- | --- | --- | --- |
|  |  |  | **(%)** | **N** | **(mm)** | **N** | **(%)** | **N** | **(°)** | **N** | **(g)** | **N** |  |
| 1 | TNAU 85S | *tms5, tms8, tms10* | 75.10 | 0.00 | 2.19 | 1.00 | 75.00 | 1.00 | 28.01 | 1.00 | 19.00 | 0.16 | 0.80 |
| 2 | TNAU 39S | *tms4, tms8, tms10* | 76.20 | 0.11 | 1.92 | 0.61 | 70.80 | 0.92 | 25.00 | 0.82 | 16.50 | 0.00 | 0.67 |
| 3 | TNAU 83S | *tms5, tms8, tms10* | 81.40 | 0.63 | 2.05 | 0.80 | 51.70 | 0.58 | 18.30 | 0.40 | 22.70 | 0.40 | 0.58 |
| 4 | TNAU 23S | *tms4, tms8, tms10* | 85.10 | 1.00 | 2.10 | 0.87 | 20.00 | 0.00 | 16.70 | 0.31 | 18.70 | 0.14 | 0.47 |
| 5 | TNAU 60S | *tms4, tms5, tms8, tms10* | 77.30 | 0.22 | 1.78 | 0.41 | 41.10 | 0.38 | 20.00 | 0.51 | 31.30 | 0.95 | 0.39 |
| 6 | TNAU 38S | *tms4, tms5, tms8, tms10* | 79.40 | 0.43 | 1.93 | 0.63 | 43.60 | 0.43 | 11.70 | 0.00 | 21.20 | 0.30 | 0.34 |
| 7 | TNAU 59S-1 | *tms5, tms8, tms10* | 78.60 | 0.35 | 1.49 | 0.00 | 49.70 | 0.54 | 11.70 | 0.00 | 32.00 | 1.00 | 0.23 |

PS:Pollen Sterility (%), PE: Panicle Exertion (%), SL: Stigma Length (mm), SE: Stigma Exertion (%), GA: Glume Angle (°), SPY: Single Plant Yield (g), N: Normalised values.
